# Supplementary material for: Molecular genetic characterization of CASEIN KINASE 1-LIKE 12 in Arabidopsis
Source: bioRxiv. 2025 Sep 19:2025.09.17.676859. Preprint. [Version 1] doi: 10.1101/2025.09.17.676859 (PMC12458278; doi:10.1101/2025.09.17.676859)
Supplement: 1 [file NIHPP2025.09.17.676859v1-supplement-1.pdf]

bioRxiv preprint doi: https://doi.org/10.1101/2025.09.17.676859; this version posted September 19, 2025. The copyright holder for this preprint (which was not certified by peer review) is the author/funder, who has granted bioRxiv a license to display the preprint in perpetuity. It is made available under aCC-BY 4.0 International license.

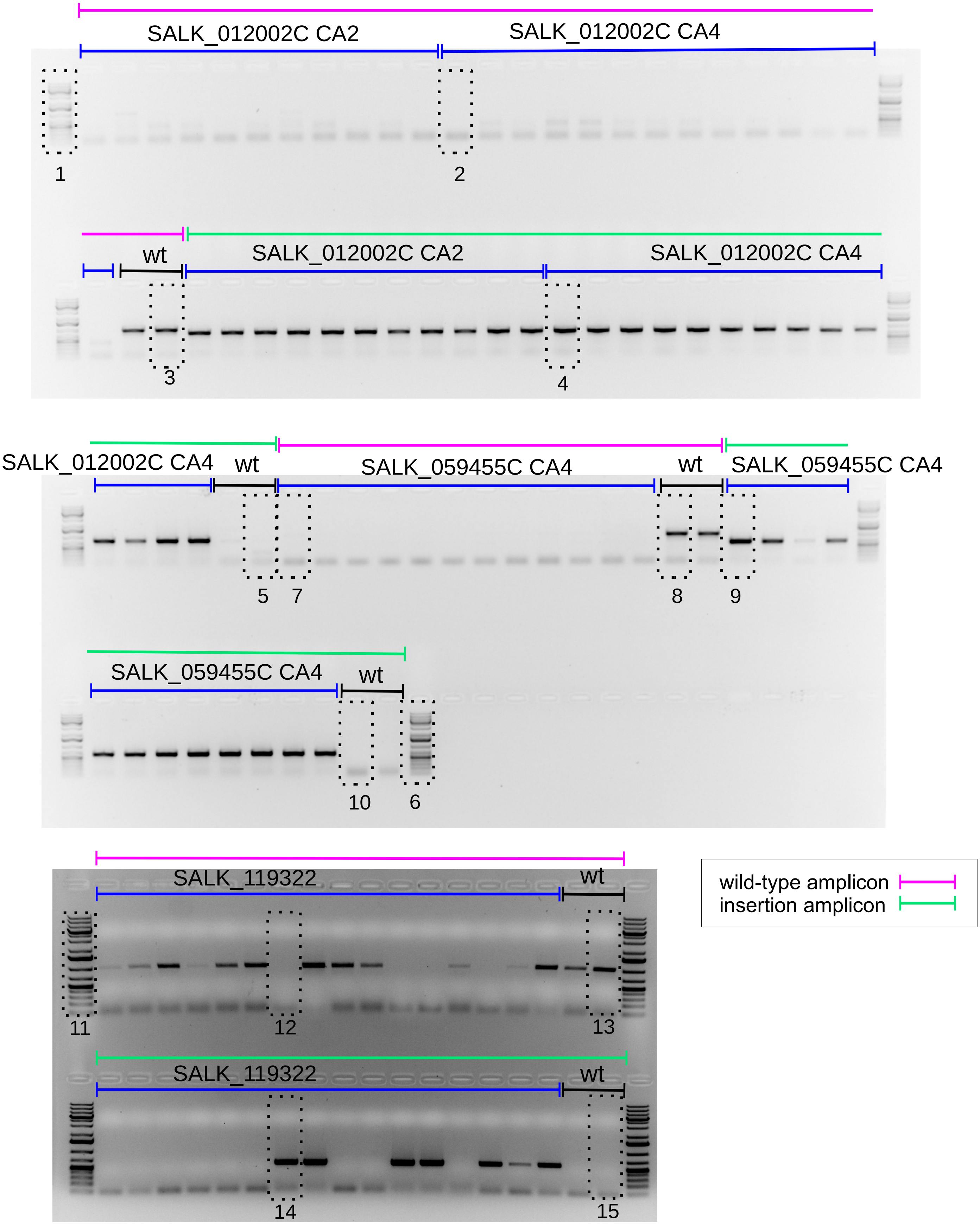


**Supplementary Figure 1: Source data for Figure 2 - Uncropped gel images**
PCR products using genomic DNA from individual plants using primers specific to wild-type or insertion sequences. Sections cropped and reassembled for Figure 2 are boxed. Numbers map to position in Figure 2.

bioRxiv preprint doi: https://doi.org/10.1101/2025.09.17.676859; this version posted September 19, 2025. The copyright holder for this preprint (which was not certified by peer review) is the author/funder, who has granted bioRxiv a license to display the preprint in perpetuity. It is made available under aCC-BY 4.0 International license.

Supplementary Table 1 - Primer Sequences

| Name | 5'-Sequence-3' | Notes |
|---|---|---|
| S059_geno_1 | GACGAGCTTGGCATCTGTTAC | Genotyping SALK_059455 |
| S059_geno_2 | TCAAAAGAATATTCCGGGACC | Genotyping SALK_059455 |
| S012_geno_1 | GTAACAGATGCCAAGCTCGTC | Genotyping SALK_012002 |
| S012_geno_2 | TCACCATAAGCCAATTTCCAC | Genotyping SALK_012002 |
| S119_geno_1 | gaatgaagatgactattgg | Genotyping SALK_119322, RT-PCR (5') |
| S119_geno_2 | CAAGTCTTCAAGACTAGGC | Genotyping SALK_119322, RT-PCR (5') |
| LBb_1.3 | ATTTTGCCGATTTCGGAAC | internal primer for SALK TDNA |
| ckl12_qRT_mid_1 | CCTTGAAGTCTGTCCTCATGC | CKL12 qRT-PCR - exons 5-6 |
| ckl12_qRT_mid_2 | GTCGATGATGTGTACCTGG | CKL12 qRT-PCR - exons 5-6 |
| ckl12_qRT_3'_1 | CATCTAGAGGCCCAATGATGC | CKL12 qRT-PCR - exons 12-13 |
| ckl12_qRT_3'_2 | GACTTGGAGAGTCTCAATGCC | CKL12 qRT-PCR - exons 12-13 |
